# The complete mitogenome of *Leptestheria brevirostris* Barnard, 1924, a rock pool clam shrimp (Branchiopoda: Spinicaudata) from Central District, Botswana

**DOI:** 10.1080/23802359.2021.1875898

**Published:** 2021-02-17

**Authors:** Arsalan Emami-Khoyi, Murphy Tladi, Tatenda Dalu, Peter R. Teske, Bettine Jansen van Vuuren, D. Christopher Rogers, Casper Nyamukondiwa, Ryan J. Wasserman

**Affiliations:** a Centre for Ecological Genomics and Wildlife Conservation, Department of Zoology, University of Johannesburg, Auckland Park, South Africa; b Department of Biological Sciences and Biotechnology, Botswana International University of Science and Technology, Palapye, Botswana; c School of Biology and Environmental Sciences, University of Mpumalanga, Nelspruit 1200, South Africa; d Kansas Biological Survey and the Biodiversity Institute, The University of Kansas, Lawrence, KS, USA; e Department of Zoology and Entomology, Rhodes University, Makhanda, South Africa

**Keywords:** Africa, Illumina next-generation sequencing, mitochondrial genome, spinicaudatan

## Abstract

Spinicaudatan clam shrimp are a widespread and diverse group of branchiopod crustaceans, yet few mitochondrial genomes have been published for this taxonomic group. Here, we present the mitogenome of *Leptestheria brevirostris* from a rock pool ecosystem in Botswana. Massively parallel sequencing of a single specimen facilitated the reconstruction of the species’ 15,579 bp circularized mitogenome. The reconstructed phylogenetic tree confirms that *L. brevirostris* forms a monophyletic group with other diplostracan branchiopods, and that these are the sister taxon to Notostraca. The mitogenome reconstructed here is the first to be reported from a leptestherid clam shrimp.

Spinicaudatan clam shrimps are a group of branchiopod crustaceans found in seasonally astatic aquatic habitats on all continents except Antarctica (Brendonck et al. 2008; Rogers 2009). These shrimps produce resting eggs capable of withstanding prolonged dry conditions between hydroperiods (Brendonck 1999). As spinicaudatan clam shrimps tend to occupy and feed in the benthic portions of pools, they are often overlooked (Brendonck et al. 2008; Rogers 2009). Here, we describe the complete mitogenome of *Leptestheria brevirostris* Barnard, 1924, a leptestherid clam shrimp that was collected from a rock pool temporary wetland in Central District, Botswana. This study forms the basis for more comprehensive phylogenetic studies to better understand the evolution of branchiopod crustaceans and their relatives.

Whole specimens of *L. brevirostris* were collected from a temporary rock pool (22°35′55.50″ S; 27°7′51.78″ E), preserved in 80% ethanol and identified using the relevant literature (Barnard 1924, 1929; Brendonck 1999). Voucher specimens from the same locality were deposited at the Kansas Biological Survey (DCR-1140). Genomic DNA of high molecular weight was extracted from a single specimen using the CTAB method (Doyle and Doyle 1987). A genomic DNA library was constructed from 1 µg of genomic DNA as template using a NEBNext Library Preparation Kit (Ipswich, MA) and sequenced on an Illumina HiSeq 4000 platform using 2 × 150 bp chemistry following the manufacturer’s instructions.

The sequencing run produced 22,933,816 paired-end sequences. The assembly of raw sequences using NovoplastyV4.2 (Dierckxsens et al. 2017) resulted in a circular genome with a total length of 15,579 bp. Annotation of the reconstructed mitogenome in MITOS webserver (Bernt et al. 2013) identified 13 protein-coding genes, 22 tRNAs, and 2 rRNAs, as is typical of all crustaceans. Instances of non-canonical start codons and truncated stop codons were observed (Jagatap et al. 2019; Monsanto et al. 2019). The protein-coding DNA sequences of the study species were aligned with those of eight closely related crustaceans in MAFFT v7.429 (Katoh and Standley 2013). A consensus Bayesian phylogenetic tree was reconstructed for the study species using BEAST2 v2.6.2 (Bouckaert et al. 2014). The program’s default settings were used, except that the substitution model was set to HKY (Hasegawa et al. 1985) with four gamma rate categories. Ten independent runs, each comprising, 500 million iterations with 150 million initial burn-in steps were executed in parallel. Final log and tree files were combined in BEAST2 LogCombiner (Rambaut and Drummond 2014), and convergence of the independent runs and effective sample size (ESS) were checked in Tracer v1.7 (Rambaut et al. 2018). The Bayesian phylogenetic tree was rooted using the anostracans *Artemia sinica* and *Streptocephalus cafer*, and visualized in FigTree v1.4 (Rambaut and Drummond 2016) (Figure 1).

**Figure 1.**
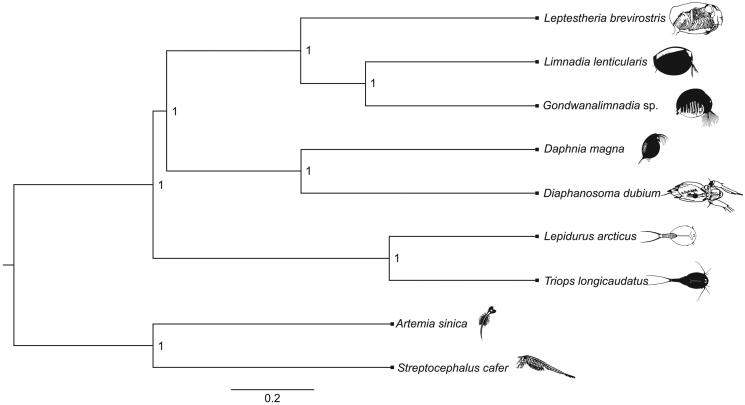
A Bayesian phylogenetic tree constructed in BEAST2 using mitogenome sequences of *Leptestheria brevirostris* (NCBI accession number MN548772) and nine other crustacean species: *Limnadia lenticularis* (NC_039394.1), *Gondwanalimnadia* sp. (MN625703.1), *Daphnia magna* (MK370029.1), *Diaphanosoma dubium* (NC_037488.1), *Lepidurus arcticus* (MK579380.1), *Triops longicaudatus* (KM516710.1), *Artemia sinica* (MK069595.1), and *Streptocephalus cafer* (MN720104.1). The numbers on the tree indicate the posterior probability of each node. The scale beneath the tree is expressed in number of substitutions per time unit.

The phylogenetic reconstruction grouped *L. brevirostris* with other spinicaudatans, and confirmed the monophyly of cladocerans and spinicaudatans and are consistent with previous studies on crustaceans mitogenomics (Luchetti et al. 2019) (Figure 1).

## Data Availability

The assembled and annotated mitogenome of the study species is available on the NCBI database (accession number MN548772; https://www.ncbi.nlm.nih.gov/search/all/?term=MN548772).
